# Tongue Pressure as a Predictor of Tongue Base Collapse in Patients with Obstructive Sleep Apnea Syndrome

**DOI:** 10.3390/biomedicines14020465

**Published:** 2026-02-19

**Authors:** Ying-Chieh Hsu, Meng-Xun Goh, Tung-Tsun Huang, Hsueh-Yu Li

**Affiliations:** 1 Department of Otolaryngology-Head & Neck Surgery, Taipei Tzu Chi Hospital, Buddhist Tzu Chi Medical Foundation, New Taipei City 23142, Taiwan; iammed91@gmail.com (Y.-C.H.); goh_1992@hotmail.com (M.-X.G.); huangtt.tw@gmail.com (T.-T.H.); 2 School of Medicine, Tzu Chi University, Hualien 97004, Taiwan; 3 Department of Otolaryngology-Head & Neck Surgery, Chang Gung Memorial Hospital, Chang Gung University, Taoyuan 33302, Taiwan

**Keywords:** obstructive sleep apnea syndrome (OSAS), tongue pressure, drug-induced sleep endoscopy (DISE), Iowa Oral Performance Instrument (IOPI)

## Abstract

**Background**: This study investigated the association between tongue strength, measured using the Iowa Oral Performance Instrument (IOPI), and upper airway collapse patterns observed during drug-induced sleep endoscopy (DISE) in patients with obstructive sleep apnea syndrome (OSAS). **Methods**: Thirty patients who underwent polysomnography, DISE, and tongue pressure measurement were retrospectively analyzed. Upper airway collapse was assessed using the VOTE classification. The tongue strength task performed using the IOPI requires participants to compress an air-filled bulb placed on the hard palate with anterior tongue to generate maximum isometric tongue pressure. Group comparisons and ordinal logistic regression with Firth’s penalized likelihood were performed to evaluate associations between tongue pressure and collapse patterns. **Results**: The participants had a mean age of 41.5 ± 12.5 years, including 27 males and 3 females. The mean tongue strength was 50.4 ± 15.3 kPa, with no significant sex-related differences. Patients with tongue strength <40 kPa showed significantly higher odds of tongue base collapse (adjusted OR 12.79, 95% CI 1.30–126.91) and epiglottic collapse (adjusted OR 54.05, 95% CI 1.66–1760.25). No significant differences were observed for velum or oropharyngeal collapse. **Conclusions**: Lower tongue strength was associated with increased likelihood of tongue base collapse during DISE. Tongue strength measurement may serve as a practical, non-invasive tool for identifying patients with reduced tongue muscle function and potential tongue-related airway obstruction.

## 1. Introduction

The prevalence of obstructive sleep apnea syndrome (OSAS) is 13% in men and 6% in women in the United States population [1]. The prevalence of OSA worldwide is estimated to be approximately 54% in the adult population [2]. There are several treatment options for OSAS, including upper airway surgery, continuous positive airway pressure (CPAP), oral appliances, and oro-myofunctional therapy [3]. Identification of the upper airway collapse level is crucial for treatment success in patients with OSAS. Various methods are available for identifying the location of an upper airway obstruction, such as nasofibroscope with Muller maneuver [4], dynamic sleep magnetic resonance imaging (MRI) and drug-induced sleep endoscopy.

Drug-induced sleep endoscopy (DISE) was first described in 1991 and has since become a widely used method for identifying the site of upper airway obstruction [5]. DISE enables direct and precise observation and evaluation of upper airway collapse under pharmacologically induced sleep status. A widely used method to score the degree of upper airway collapse during DISE is VOTE classification [6]. VOTE classification represents the velum, oropharynx (including tonsil), tongue base, and epiglottis. The degree of airway obstruction is classified as no obstruction (0, <50%), partial obstruction (1, 50–75%), and complete obstruction (2, >75%).

Despite its effectiveness, DISE is an invasive and costly procedure, and its findings can be affected by the anesthetic drug and its concentration. Heo et al. found that prolonging the DISE procedure, and thereby increasing the total dose of Midazolam, could increase the number of obstruction sites and the obstruction severity [7].

Poor effectiveness of upper airway muscles is thought to contribute to the pathogenesis of OSAS [8]. A recent study showed that oro-myofunctional (OMT) therapy could improve sleep apnea severity by increasing upper airway muscle tone and responsiveness. Guimaraes et al. observed a significant reduction in AHI during the REM stage, daytime sleepiness, and sleep quality score in an OMT group. Among all the upper airway dilator muscles, the genioglossus muscle is considered the most important muscle that compensates for upper airway collapse in OSAS patients. However, there is a lack of studies examining its function and the role of the tongue base in OSAS patients undergoing DISE. Therefore, we hypothesize that tongue strength in OSAS patients may be associated with the level of anatomical collapse observed during DISE.

## 2. Materials and Methods

### 2.1. Ethics Approval

This study was conducted in accordance with the Declaration of Helsinki and approved by the Institutional Review Board of Taipei Tzu Chi Hospital (IRB No. 14-IRB007).

We retrospectively reviewed medical records of patients aged 18 years or older who underwent standard polysomnography (PSG), drug-induced sleep endoscopy (DISE) and tongue pressure measurement. All the participants completed the Epworth Sleepiness Scale in the sleep laboratory on the night of the polysomnography. The Iowa Oral Performance Instrument (IOPI) (model 3.1; IOPI Medical LLC, Woodinville, WA, USA) was used to measure the maximum tongue strength. The subject was seated in the upright position. The IOPI bulb was placed on the central anterior tongue directly behind the front teeth. All participants were instructed to press the bulb upward against the palate as hard as possible, usually for a brief period (1–2 s), and this was repeated for three trials, with the highest-pressure value (in kPa) recorded as the maximum tongue strength. All tongue strength measurements were performed by the same investigator (Hsu YC). Patients with an apnea–hypopnea index (AHI) of less than 5 or with missing data were excluded from the study, as were those who had undergone tongue/tongue base surgery before. Ultimately, 30 patients (27 males and 3 females) were included in our study.

DISE was conducted in a silent operating room. Artificial sleep was induced with intravenous administration of propofol via a target-controlled infusion (TCI) system. The bispectral index (BIS) was used to monitor the depth of anesthesia level and is crucial in DISE by optimizing the simulation of natural sleep and improving the reliability of upper airway assessment. The BIS index was kept between 50 and 70 to avoid oversedation [9]. DISE findings, recorded previously as videos, were retrospectively reviewed and scored according to the VOTE classification. The interpretation was conducted by the agreement of two different surgeons, who worked independently of each other and were blinded to the clinical data.

### 2.2. Statistical Analysis

Continuous variables are presented as mean ± standard deviation or median (interquartile range), as appropriate. Categorical variables are presented as frequencies and percentages. Group comparisons were performed using Student’s *t*-test or the Mann–Whitney U test for continuous variables and chi-square or Fisher’s exact test for categorical variables. In our cohort, there were only four epiglottic collapse cases. In such instances, standard maximum likelihood estimation (MLE) often fails to converge. Therefore, we chose Firth’s penalized likelihood approach to get more reliable odds ratios (ORs) and confidence intervals for the association between IOPI tongue pressure and the site of collapse. Multivariable models were adjusted for age, body mass index (BMI), and apnea–hypopnea index (AHI). Statistical analyses were performed using SAS version 4.1.0, and a two-sided *p*-value < 0.05 was considered statistically significant.

## 3. Results

The participants had a mean age of 41.5 ± 12.5 years and a mean BMI of 27.5 ± 4.4 kg/m^2^. The mean AHI was 43.1 ± 28.8 per hour. The distribution of VOTE classification was as follows: velopharyngeal obstruction (V0:6.7%, V2:93.3%), oropharyngeal lateral wall obstruction (O0:30%, O1:26.7%, O2:43.3%), tongue base obstruction (T0:53.3%, T1:30%, T2:16.7%), epiglottic collapse (E0:86.7%, E1:3.3%, E2:10%). (Table 1).

The mean IOPI tongue pressure was 50.4 ± 15.3 kPa, and there was no significant difference observed between genders in terms of tongue pressure, as indicated by both mean and median values (Table 2).

We categorized the patients into two groups based on their IOPI tongue pressure: IOPI < 40 kPa and IOPI ≥ 40 kPa. The normal range of tongue strength measured by IOPI in healthy adults ranged from 40 to 80 kPa. The 40 kPa cutoff was selected as an exploratory threshold aligning with the prior literature [10] and our clinical experience regarding reduced tongue strength in OSA populations in Taiwan, rather than a universally acknowledged cutoff value. Then, we compared the variables between the two groups to determine whether there were any statistically significant differences. The results of this analysis are presented in Table 3.

Except for the tongue base and epiglottic collapse levels, no significant differences were observed between the two groups in terms of all variables, including age, gender, BMI, and AHI.

To account for other potential confounding factors, we used an ordinal logistic regression model with Firth’s penalized likelihood approach to investigate the association between IOPI groupings and VOTE classifications. This approach is particularly useful for addressing issues related to small sample sizes and reducing bias in parameter estimates. For tongue base obstruction, the crude model showed a statistically significant association (*p* = 0.019), with an odds ratio (OR) of 9.44 and a 95% confidence interval (CI) of 1.44 to 61.81 in the IOPI < 40 kPa group compared to the IOPI ≥ 40 kPa group. After adjusting for potential confounders, the OR increased to 12.79 (95% CI 1.30,126.91). For epiglottis obstruction, the crude model also showed a statistically significant association (*p* = 0.012), with an OR of 27.42 (95% CI 2.09,359.52) in the IOPI < 40 kPa group compared to the IOPI ≥ 40 kPa group. After adjusting for potential confounders, the OR increased to 54.05 (95% CI 1.66, 1760.25), with a *p*-value of 0.025. These findings indicate that lower tongue pressure was independently associated with increased severity of tongue base collapse and, to a lesser extent, epiglottic collapse (Table 4).

## 4. Discussion

The major findings from this study support the concept that reduced tongue muscle strength represents a functional endotype contributing to upper airway collapsibility in OSAS.

The tongue is a commonly collapsed site in OSAS patients, accounting for 58% of obstruction cases [11]. The genioglossus muscle (GG), which is the largest upper airway dilator muscle, plays an important role in maintaining upper airway patency during sleep by depressing and protruding the tongue [12]. The main function of the GG is tongue depression and protrusion. Clinicians use DISE, dynamic magnetic resonance imaging (MRI) or computed tomography to predict tongue obstruction. However, some patients cannot fall asleep during dynamic MRI due to its loud noise. The DISE procedure is an invasive and expensive procedure and needs to be performed in an operating room. At present, we still lack an easy, non-invasive tool to predict tongue obstruction in OSAS patients in clinical practice.

In our study, we investigated the relationship between tongue strength and tongue collapse severity in OSAS patients. We conducted a statistical analysis of data obtained from measuring tongue strength and found a significant association between tongue pressure measured by IOPI and the likelihood of tongue base and epiglottic collapse in OSAS patients. Specifically, when the tongue pressure was less than 40 kPa, there was a significantly increased likelihood of tongue base and epiglottic collapse, with odds ratios of 12.79 (*p* = 0.03) and 54.05 (*p* = 0.025), respectively. Our findings are consistent with previous studies by Evangelisti et al. and O’Connor-Reina et al., which also reported lower tongue strength and peak pressure in children with sleep-disordered breathing and OSAS patients, respectively, as measured by the IOPI, compared to healthy individuals [13,14]. Interestingly, our results align with those of O’Connor-Reina et al., who found a significant correlation between IOPI scores and tongue collapse during DISE. However, there is no precise IOPI score to predict tongue obstruction in OSAS patients. In our study, we discovered that the likelihood of tongue and epiglottic collapse would increase when the IOPI score < 40 kPa. To our knowledge, this is the first study to report the use of an IOPI tongue pressure score of less than 40 kPa as a potential tool to predict tongue base obstruction in OSAS patients. This finding has important clinical implications for predicting and managing tongue collapse severity in OSAS patients and may inform the development of non-invasive, cost-effective tools for screening and diagnosing tongue obstruction in this population. However, although a statistical association was observed between lower IOPI values and epiglottic collapse, the small number of epiglottic obstruction cases and the wide confidence intervals indicate substantial uncertainty. These findings should therefore be interpreted with caution as exploratory.

Orofacial myofunctional therapy (OMT) has been shown to improve OSA severity by enhancing upper airway muscle tone and neuromuscular responsiveness. In a randomized controlled study, Guimarães et al. [15] demonstrated significant reductions in AHI, particularly during REM sleep, along with improvements in daytime sleepiness and sleep quality following OMT, suggesting a sustained augmentation of pharyngeal dilator function, especially involving the genioglossus muscle. These findings are supported by a systematic review and meta-analysis by Camacho et al. [16], which reported mean reductions in AHI of approximately 50% in adults and 62% in children receiving OMT. As interest grows in identifying patients most likely to benefit from OMT, objective assessment of tongue muscle function has gained attention. Suzuki M et al. [17] demonstrated that OSA patients with low tongue pressure benefit from 6-month oro-myofunctional therapy, showing significant improvements in tongue pressure and AHI. Collectively, these findings suggest that tongue pressure measurement may serve as a practical adjunct for identifying the endotype of OSA patients with reduced tongue muscle function. The identification of functional endotypes in OSA patients is crucial for personalized and precision treatment. Based on our research findings, we suggest tongue pressure measurement may serve as a rapid, non-invasive screening tool to identify patients at higher likelihood of tongue base collapse (IOPI score of less than 40 kPa), thereby informing the need for DISE, prioritization of myofunctional therapy, pharmacotherapy [18] or consideration of tongue-targeted surgical interventions [19,20].

Several limitations should be acknowledged. First, the relatively small sample size limits generalizability and resulted in wide confidence intervals, particularly for epiglottic collapse. Therefore, findings related to epiglottic obstruction should be interpreted cautiously. Future studies with larger cohorts are required to validate these observations. Second, inclusion was limited to patients who underwent both DISE and tongue pressure measurement, which may introduce selection bias and limit applicability to broader OSA populations. Third, this study did not assess the relationship between tongue strength and treatment outcomes; therefore, while an association between tongue pressure and upper airway collapse was observed, future prospective studies are required to determine whether interventions that improve tongue strength translate into meaningful clinical benefits.

## 5. Conclusions

This study investigates the correlation between tongue strength and the anatomical collapse level observed during DISE in patients with OSAS. The findings showed that the IOPI is a practical and valuable tool for measuring tongue pressure, and it can assist in identifying the “low muscle tone” endotype in OSAS. Moreover, our study’s results indicate that IOPI levels below 40 kPa were associated with a higher likelihood of tongue base collapse observed during DISE. These results have potential clinical implications for managing the severity of tongue collapse in OSAS patients and may inform the future development of non-invasive and cost-effective tools for screening for tongue obstruction in this population.

## Figures and Tables

**Table 1:** Demographics of the sample population.

	n (%)	Mean	SD	Min	Max	Median (IQR)
**Age**		41.5	12.5	25	68	39.0 (30.0–52.0)
**Sex**						
** Men**	27 (90%)					
** Female**	3 (10%)					
**BMI**		27.5	4.4	22.0	38.4	26.9 (24.0–29.1)
**ESS**		10.3	5.8	1.0	21.0	9.0 (6.0–15.0)
**IOPI**		50.4	15.3	18.0	74.0	53.0 (41.5–62.9)
** IOPI < 40 (N = 6)**		26.8	6.5	18.0	36.8	26.6 (23.0–30.0)
** IOPI > 40 (N = 24)**		56.9	9.4	40.0	74.0	55.6 (51.0–66.0)
**Tongue tie**		4.1	1.0	0.5	5.0	4.0 (4.0–4.5)
**AHI**		43.1	28.8	6.1	95.9	34.4 (22.7–66.8)
**Min O2**		76.6	9.7	58.0	90.0	78.0 (66.0–85.0)
**V: velum**						
** 0**	2 (6.7%)					
** 1**	0 (0%)					
** 2**	28 (93.3%)					
**O: oropharynx**						
** 0**	9 (30%)					
** 1**	8 (26.7%)					
** 2**	13 (43.3%)					
**T: tongue base**						
** 0**	16 (53.3%)					
** 1**	9 (30%)					
** 2**	5 (16.7%)					
**E: epiglottic**						
** 0**	26 (86.7%)					
** 1**	1 (3.3%)					
** 2**	3 (10%)					

Abbreviation: SD, standard deviation; BMI, body mass index; ESS, Epworth sleepiness scale; IOPI, Iowa Oral Performance Instrument; AHI, apnea–hypopnea index.

**Table 2:** IOPI compared by gender.

IOPI	Male	Female	*p*
Mean ± SD	49.42 ± 14.88	58.93 ± 19.58	0.318
Median (IQR)	53.0 (43.0–57.9)	66.0 (36.8–74.0)	0.314

Abbreviation: SD, standard deviation; IOPI, Iowa Oral Performance Instrument; IQR, interquartile range. Note: *p*-values were calculated using Student’s *t*-test for variables presented as mean ± SD and the Mann–Whitney *U* test for variables presented as median (IQR). A two-sided *p*-value < 0.05 was considered statistically significant.

**Table 3:** Comparison of the collapse degree based on IOPI levels.

	IOPI < 40 (N = 6)	IOPI ≥ 40 (N = 24)	*p*
**Age**	39.5 ± 17.2	42.1 ± 12	0.673
**Sex**			0.595
** Female**	1 (16.7%)	2 (9.1%)	
** Male**	5 (83.3%)	20 (90.9%)	
**BMI**	26.9 ± 5.6	27.8 ± 4.3	0.651
**ESS**	9 ± 6.2	10.4 ± 6	0.629
**AHI**	39.3 (34.1,55.4)	33.8 (24.5,81.8)	0.573
**Min O2**	75.7 ± 10.7	75.8 ± 9.7	0.970
**V: velum**			
** 0**	0 (0%)	2 (9.1%)	0.443
** 2**	6 (100%)	20 (90.9%)	
**O: oropharynx**			
** 0**	2 (33.3%)	7 (31.8%)	0.858
** 1**	1 (16.7%)	6 (27.3%)	
** 2**	3 (50%)	9 (40.9%)	
**T: tongue base**			
** 0**	1 (16.7%)	14 (63.6%)	0.040
** 1**	2 (33.3%)	6 (27.3%)	
** 2**	3 (50%)	2 (9.1%)	
**E: epiglottic**			
** 0**	3 (50%)	21 (95.5%)	0.002
** 1**	0 (0%)	1 (4.5%)	
** 2**	3 (50%)	0 (0%)	

We divided these 30 patients into two groups: IOPI < 40 kPa and IOPI > 40 kPa. All the variables between these two groups showed no significant difference, except collapse in epiglottic level. Abbreviation: BMI, body mass index; ESS, Epworth sleepiness scale; IOPI, Iowa Oral Performance Instrument; AHI, apnea–hypopnea index. Statistical comparisons for continuous variables were performed using Student’s *t*-test (age, BMI, ESS, and minimum O_2_) or the Mann–Whitney *U* test (AHI), according to data distribution. Fisher’s exact test was used for categorical variables (sex and VOTE classification) because of small cell counts (N < 5).

**Table 4:** The association of IOPI and VOTE using the ordinal logistic regression model with Firth’s penalized likelihood approach.

	IOPI < 40	IOPI ≥ 40	*p*-Value
	Odds Ratio (95% CI)	Reference Group	
**V**			
Crude model	1.59 (0.05,46.79)	1	0.790
Adjusted model ^†^	2.02 (0.10,40.35)	1	0.644
**O**			
Crude model	1.21 (0.23,6.53)	1	0.823
Adjusted model ^†^	1.46 (0.19,11.49)	1	0.719
**T**			
Crude model	9.44 (1.44,61.81)	1	0.019
Adjusted model ^†^	12.79 (1.30,126.91)	1	0.030
**E**			
Crude model	27.42 (2.09,359.52)	1	0.012
Adjusted model ^†^	54.05 (1.66,1760.25)	1	0.025

^†^ The results were analyzed using multiple ordinal logistic regression after adjusting for age, sex, and BMI.

## Data Availability

The data that support the findings of this study are available on request from the corresponding author.

## Abbreviations

The following abbreviations are used in this manuscript:

IOPI	Iowa Oral Performance Instrument
DISE	drug-induced sleep endoscopy
OSAS	obstructive sleep apnea syndrome
CPAP	continuous positive airway pressure
MRI	magnetic resonance imaging
OMT	oro-myofunctional therapy
AHI	apnea–hyponea index
PSG	polysomnography
TCI	target-controlled infusion
BIS	bispectral index
BMI	body mass index
GG	genioglossus muscle

## References

[1] Peppard P.E., Young T., Barnet J.H., Palta M., Hagen E.W., Hla K.M. (2013). Increased prevalence of sleep-disordered breathing in adults. Am. J. Epidemiol..

[2] de Araujo Dantas A.B., Goncalves F.M., Martins A.A., Alves G.Â., Stechman-Neto J., Correa C.D.C., Santos R.S., Nascimento W.V., de Araujo C.M., Taveira K.V.M. (2023). Worldwide prevalence and associated risk factors of obstructive sleep apnea: A meta-analysis and meta-regression. Sleep Breath..

[3] Gottlieb D.J., Punjabi N.M. (2020). Diagnosis and management of obstructive sleep apnea: A review. JAMA.

[4] Terris D.J., Hanasono M.M., Liu Y.C. (2000). Reliability of the Muller maneuver and its association with sleep-disordered breathing. Laryngoscope.

[5] Croft C., Pringle M. (1991). Sleep nasendoscopy: A technique of assessment in snoring and obstructive sleep apnoea. Clin. Otolaryngol. Allied Sci..

[6] Kezirian E.J., Hohenhorst W., de Vries N. (2011). Drug-induced sleep endoscopy: The VOTE classification. Eur. Arch. Oto-Rhino-Laryngol..

[7] Heo S.J., Park C.M., Kim J.S. (2014). Time-dependent changes in the obstruction pattern during drug-induced sleep endoscopy. Am. J. Otolaryngol..

[8] Susarla S.M., Thomas R.J., Abramson Z.R., Kaban L.B. (2010). Biomechanics of the upper airway: Changing concepts in the pathogenesis of obstructive sleep apnea. Int. J. Oral. Maxillofac. Surg..

[9] Lo Y.L., Ni Y.L., Wang T.Y., Lin T.Y., Li H.Y., White D.P., Lin J.R., Kuo H.P. (2015). Bispectral index in evaluating effects of sedation depth on drug-induced sleep endoscopy. J. Clin. Sleep. Med..

[10] Utanohara Y., Hayashi R., Yoshikawa M., Yoshida M., Tsuga K., Akagawa Y. (2008). Standard values of maximum tongue pressure taken using newly developed disposable tongue pressure measurement device. Dysphagia.

[11] Lee E.J., Cho J.H. (2019). Meta-analysis of obstruction site observed with drug-induced sleep endoscopy in patients with obstructive sleep apnea. Laryngoscope.

[12] Cori J.M., O’Donoghue F.J., Jordan A.S. (2018). Sleeping tongue: Current perspectives of genioglossus control in healthy individuals and patients with obstructive sleep apnea. Nat. Sci. Sleep.

[13] Evangelisti M., Martella S., Barreto M., Villa M.P. (2017). Tongue strength evaluation in children with and without sleep disordered breathing. Eur. Resp. J..

[14] O’Connor-Reina C., Plaza G., Garcia-Iriarte M.T., Ignacio-Garcia J.M., Baptista P., Casado-Morente J.C., De Vicente E. (2020). Tongue peak pressure: A tool to aid in the identification of obstruction sites in patients with obstructive sleep apnea/hypopnea syndrome. Sleep Breath..

[15] Guimarães K.C., Drager L.F., Genta P.R., Marcondes B.F., Lorenzi-Filho G. (2009). Effects of oropharyngeal exercises on patients with moderate obstructive sleep apnea syndrome. Am. J. Respir. Crit. Care Med..

[16] Camacho M., Certal V., Abdullatif J., Zaghi S., Ruoff C.M., Capasso R., Kushida C.A. (2015). Myofunctional therapy to treat obstructive sleep apnea: A systematic review and meta-analysis. Sleep.

[17] Suzuki M., Okamoto T., Akagi Y., Matsui K., Sekiguchi H., Satoya N., Inoue Y., Tatsuta A., Hagiwara N. (2021). Efficacy of oral myofunctional therapy in middle-aged to elderly patients with obstructive sleep apnoea treated with continuous positive airway pressure. J. Oral. Rehabil..

[18] Lee Y.C., Lu C.T., Chuang L.P., Lee L.A., Fang T.J., Cheng W.N., Li H.Y. (2023). Pharmacotherapy for obstructive sleep apnea—A systematic review and meta-analysis of randomized controlled trials. Sleep Med. Rev..

[19] Hsin L.J., Lee Y.C., Lin W.N., Lu Y.A., Lee L.A., Tsai M.S., Cheng W.N., Chiang Y.T., Li H.Y. (2022). Transoral Tongue Suspension for Obstructive Sleep Apnea—A Preliminary Study. J. Clin. Med..

[20] Lu Y.A., Wang C.J., Chiang Y.T., Li H.Y. (2022). Volumetric Changes after Coblation Ablation Tongue (CAT) in Obstructive Sleep Apnea Patients. J. Clin. Med..

